# Contemporary practice of standardised bedside teaching rounds

**DOI:** 10.1111/tct.13228

**Published:** 2020-07-28

**Authors:** Clarence Haddon Mullins, Adam Roderick, Jill Deaver, James Willig

**Affiliations:** ^1^ University of Alabama at Birmingham School of Medicine Birmingham Alabama USA; ^2^ UABTeach University of Alabama at Birmingham Birmingham Alabama USA; ^3^ Lister Hill Library of the Health Sciences University of Alabama at Birmingham Birmingham Alabama USA; ^4^ Department of Infectious Disease University of Alabama School of Medicine Birmingham Alabama USA

## Abstract

**Background:**

The purpose of this article is to review the extant literature on bedside teaching rounds within the context of ward rounds performed with the entire clinical team at the patient’s bedside, and to assess the effects of standardisation of this process on patient and learner satisfaction, as well as other duty‐hour restrictions and patient care metrics in the academic inpatient setting. Ultimately, the intent of this review is to inform faculty development sessions for educators on the benefits and challenges of standardised rounding protocols.

**Methods:**

We performed a search of PubMed, Scopus and CINAHL databases (from 2003 to August 2019). Randomised, controlled trials, pre‐ and post‐interventional studies and cohort studies, in English, were eligible for inclusion. Two reviewers independently searched, screened and analysed the studies, and a narrative synthesis was performed. Articles were evaluated methodologically using the Medical Education Research Quality Study Instrument (MERQSI).

**Results:**

Five articles were included, with one randomised controlled trial, three cohort studies, and one pre‐ and post‐interventional study. The collective MERSQI score for the studies was 12.3. Patient satisfaction increased uniformly across studies when standardised practices were used. Attempts to improve learner satisfaction, however, achieved mixed results. In addition, the time of bedside rounds was found to decrease with standardised interventions overall.

**Conclusion:**

In light of generally positive albeit limited evidence for standardised rounding practices, faculty development initiatives might use these data to inform and educate faculty members regarding the use of standardised protocols for bedside rounds.

1

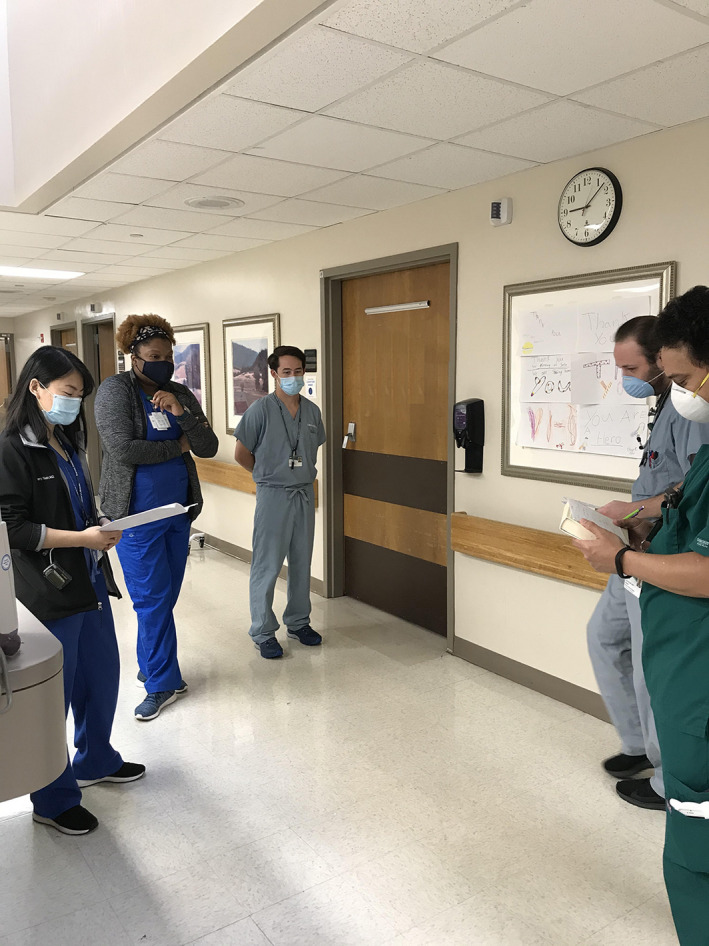

## 2 INTRODUCTION

Bedside rounds, or rounds that take place in the presence of the patient at the bedside, have become increasingly replaced with ‘hallway’ or ‘conference table’ rounds in the USA.^1^ As the rates of bedside encounters decline, educators have become increasingly concerned about the potential adverse effects such trends may have on the future of medical education and the patient experience.^2^ In addition, work‐hour limits and increasing trainee and faculty member pressure to combine clinical and educational experience have created a system where trainee satisfaction and time demands are often at odds with each other.^3^

Although numerous studies investigating the factors associated with successful rounds and ways in which to meet these challenges have been performed, little is known about how rounds might be standardised across the graduate medical education (GME) continuum, and how such standardisation might impact resident education or patient satisfaction. The purpose of this article is to provide a review of the literature reporting standardised practices for bedside rounds, and their effects on patients and learners, in a contemporary time frame affected by duty‐hour restrictions and other time‐based productivity metrics. In doing so, the information from this review might be used to inform faculty development sessions regarding the potential benefits and challenges faced by educators when attempting to standardise bedside rounds.

… little is known about how rounds might be standardised across the graduate medical education …

The review team included an internal medicine physician, a liaison librarian with the School of Medicine, an educator in the undergraduate setting with a focus on pedagogy and a medical student focused on academic research.

We conducted a review of the following databases for papers published between 2003 and August 2019: PubMed (via the National Center for Biotechnology Information, NCBI), Scopus (via Elsevier) and the Cumulative Index to Nursing and Allied Health Literature (CINAHL) (via EBSCO Information Services). The search strategy was specific to each database, but key terms used included ‘bedside’, ‘teaching’, ‘rounds’, ‘education’, ‘curriculum’, ‘students’, ‘residents’ or ‘medical’. The goal was to define rounds as broadly as possible while ensuring that education was a focus to some extent, as opposed to some interprofessional rounds that may be strictly clinical as opposed to pedagogical. The search sought to include any manuscript mentioning ‘teaching rounds’, ‘bedside rounds’ or ‘patient room or bedside’ and ‘teaching’. Randomised controlled trials, prospective cohort and retrospective cohort studies were eligible for inclusion. Criteria for exclusion were specified as studies that did not involve learners, educators and patients together, did not provide validated, qualitative or quantitative methods, were published prior to the 2003 duty‐hour regulation changes in the USA and all commentaries, reviews or opinions. This time frame was determined in order to limit the potential confounding of learner satisfaction prior to duty‐hour restriction implementation, as well as to ensure that the recommendations made are amenable to current regulations.

Two reviewers independently screened 451 citations for compliance with inclusion criteria, based on the title, abstract review and full‐text review of 235 articles that met the above criteria. In addition, the references for all papers not initially excluded were reviewed to ensure a more complete scope of the review. We then extracted all relevant data on the characteristics of participants, interventions and outcome measures using a structured data extraction form. The majority of studies were excluded for not attempting to standardise the definition or process of bedside rounds, for not including learners or for being a review article without providing empirical data.

Furthermore, studies were assessed for methodological quality using the Medical Education Research Study Quality Instrument (MERSQI), providing each publication a score on an ordinal scale with a range from 5 to 18. MERSQI is a tool with proven validity and reliability, designed to assess the methodological quality of observational, experimental and quasi‐experimental studies in medical education using six domains: study design, sampling, type of data, validity, data analysis and outcomes.^4^

## 3 RESULTS

The search yielded five articles. The average MERSQI score for the articles included was 12.3 (Table 1). Of the studies included in this review there was one randomised controlled trial,^5^ one retrospective cohort study,^6^ and three prospective cohort studies.^7^, ^8^, ^9^ Observed elements of standardisation included adherence rates, standardisation components, the assessment of learner satisfaction, the assessment of patient satisfaction and the time burden of the bedside rounds.

**Table 1:** Descriptions of included studies

Citation	Design/Method	Location	Sample/Setting	Elements of Standardisation	Outcomes	MERSQI
Bennett et al., 2017^7^	Quasi‐experimental design	USA	188 residents at an academic hospital 19 complete rounds observed	Morning huddles, bedside rounds, diagnostic ‘time outs’, day‐of‐discharge rounds, post‐discharge follow‐up rounds	Significantly improved resident satisfaction in education and patient care	10.0
Calderon et al., 2014^6^	Retrospective cohort	USA	17,375 patients in an academic hospital	‘Rounding‐in‐flow’ Each patient seen at bedside, followed by documentation and order placement before seeing to the next patient. Two interns alternate between pre‐rounding and bedside presentations	Significantly improved resident duty hour compliance and intern work hours	12.5
Gonzalo et al., 2010^8^	Controlled before‐and‐after study	USA	16 residents and 32 interns 1798 total rounding encounters	Preparation, orientation, bedside rounds, bedside teaching and debriefing	Significantly improved patient preference for bedside rounds	12.0
Monash et al., 2017^5^	Cluster randomised controlled trial	USA	500 bed urban hospital, 1200 hospital inpatients 3758 patient encounters	Pre‐round huddle, bedside rounds, nurse integration, real‐time order entry, whiteboard updates	Significantly improved overall patient satisfaction and shortened rounding time	14.0
Southwick et al., 2014^9^	Interrupted‐time‐series study	USA	16 multidisciplinary medicine teams observed for 11 months in an academic hospital	Introduction of job descriptions (faculty member, house staff, medical student, nurse, pharmacist and case manager), identification of customer–supplier relationships, communication protocols (Subjective, Objective, Assessment, Plan and Situation, Background, Assessment, Recommendation), weekly feedback	Significantly improved 30‐day readmission rate and length of stay	13.0

Overall, adherence rates to study‐specific standardisation protocols ranged from 41 to 81%. Only two studies reported adherence to individual components of the recommended systems. Interestingly, the two components shared by each of those studies, ‘pre‐round huddle’ (or briefing) and ‘bedside rounds’, also had the highest adherence rates in each study, with rates of 89 and 95%, respectively, reported by Bennett et al., and 78 and 53% reported by Monash et al.^5^, ^7^

### 3.1 Learner satisfaction

Southwick et al. reported that students in the experimental group felt more satisfied with rounds, more integrated and rated teachers more highly in bi‐weekly surveys scored on a five‐point Likert scale.^9^ Residents in the experimental group study also had increased overall satisfaction and thought that the rounds were more efficient. Validity and reliability evidence for these surveys were not reported.

Bennett et al. evaluated the programme by qualitative ethnographic observation, semi‐structured interviews and focus groups, as well as with a standard departmental survey.^7^ Residents in the intervention group rated the rotation significantly higher, reported an increase in the regular occurrence of teaching during rounds and also reported that faculty members were more invested in the educational aspects of the rounds. In addition, all residents felt that each individual component (morning huddle, bedside rounds, diagnostic ‘time out’, day‐of‐discharge rounds and post‐discharge follow‐up) was effective, and 100% of residents reported that they would continue to use components from the system in the future.^7^

Of the two studies that reported significantly decreased satisfaction among learners, the study by Monash et al. demonstrated decreased satisfaction overall with rounds, and interestingly employed the same survey used in the study by Southwick et al.^5^ The study by Gonzalo et al. used a previously piloted survey and suggested that residents believe bedside rounds to be more educational, but did not demonstrate a significant preference overall towards bedside rounds.^8^

Although previously identified barriers to the regular conduction of formal teaching rounds include a lack of satisfaction among learners,^10^ the overall conclusion from these studies support increased satisfaction with standardised bedside teaching rounds. Even though one study reported decreased learner satisfaction with standardised beside rounds,^5^ it is possible that the decreased satisfaction stemmed from a false perception that standardisation increased the duration of the rounds. Additionally, previous work has demonstrated that learners with prior exposure to bedside rounds were more likely to prefer them, so it is possible that the dissatisfaction represented here reflects the institution of a new process, and that with time these learners would also come to prefer standardised bedside teaching rounds.^11^

… the overall conclusion from these studies support increased satisfaction with standardised bedside teaching rounds

### 3.2 Patient satisfaction

Three studies evaluated the impact that standardised rounds had on patient satisfaction.^5^, ^8^, ^9^ In two trials, patients were found to significantly prefer bedside rounds,^5^, ^8^ whereas one trial showed no difference.^9^ In the study performed by Gonzalo et al., patients reported significantly increased satisfaction with bedside rounds and perceived that teams spent significantly more time with them after the intervention.^8^ In the study by Monash et al., patients in the intervention arm were significantly more satisfied with patient rounds and felt more cared for by their teams.^5^

These studies also support previous data that suggest patients prefer bedside rounds as opposed to conference room or ‘table rounds’,^10^ which in contemporary settings where patient satisfaction is gaining increasing importance suggests an institutional benefit to promoting standardised bedside rounds. They also demonstrate that standardisation can increase the frequency of bedside rounds,^8^ and that standardisation further increases patient satisfaction compared with previous bedside rounds.^5^

… patients prefer bedside rounds as opposed to conference room or ‘table rounds’ …

### 3.3 Timeliness of rounds

Three of the studies reported outcomes related to the timeliness or efficiency of the rounds, with Calderon et al. reporting an increase in the completion of discharge orders before 09:00 h.^6^ In addition, Monash et al. and Southwick et al. both reported a decrease in the overall time spent on rounds.^5^, ^9^ Notably, the learners studied by Monash et al. reported a perception of longer lasting rounds,^5^ even though, objectively, the rounds were significantly shorter. Possibly, this perception is mediated by a more global dissatisfaction of standardised rounds among trainees, secondary to decreased autonomy or an increase in the cognitive load required to conform to new practices.

Although often cited as a barrier to bedside rounds, this review of the literature has shown this perception of time constraints to be erroneous.^10^ In fact, the literature examined in this review suggests that standardisation can decrease the time taken to complete bedside rounds,^5^, ^9^ with a beneficial impact on efficiency that in turn can impact discharge timing and free learner time to pursue other activities important to their professional development or patient care.

… standardisation can decrease the time taken to complete bedside rounds …

## 4 RECOMMENDATIONS

The clear lack of randomised controlled trials or rigorous qualitative information on this topic, and indeterminate potential effects of confounding or bias, leave limited room for general recommendations beyond the need for more research. Thus, we have relayed our conclusions and personal recommendations after instituting standardised bedside rounds into our practice over the past 5 years, and place an emphasis on three components as a focus: pre‐round huddles; rounds at the bedside; and the inclusion of nursing staff and other providers (e.g. pharmacists or case managers) if possible. Pre‐round meetings among undergraduate and graduate trainees in preparation for rounds (pre‐round huddle) will help to organise the content presented to patients and educators and will impact efficiency favourably. Bedside presentations and discussions are viewed favourably by the patients, allow learners to engage in clinical reasoning discussions and review relevant physical exam findings, and engage all stakeholders in crafting a plan for care. The inclusion of nursing staff encourages interdisciplinary communication and ensures that care decisions are executed fluidly and efficiently, to the benefit of the patient.

The inclusion of nursing staff encourages interdisciplinary communication and ensures that care decisions are executed fluidly and efficiently …

### 4.1 Faculty development

Perhaps somewhat uniquely, the authors used the information gathered in this review to develop a podcast for a locoregional faculty development series on bedside teaching. Although the popularity of this asynchronous dissemination technique is growing, we certainly do not expect most faculty development exercises to go to such lengths and, as such, developed in‐person educational sessions as well. In‐person sessions ensure uniform distribution and may contribute to a shared mental model among educators. In addition, these sessions allow for interaction or question‐and‐answer style learning that may further help to reassure those hesitant to adopt new practices. Asynchronous methods, however, provide educators with more control over the content and timing of delivery, and may improve retention over time or the adoption of specific, highly convincing aspects of standardised protocols (Box 1).

Box 1 **Leading a faculty development session on standardised bedside teaching rounds**

Many faculty members may already have experience with bedside rounds but may be less familiar with evidence for standardisation or examples of attempts to do so.

Session leaders may refer to articles detailed in this review for supporting evidence and further examples.

**Explore**

- What experience do members have with bedside rounds?
- How might standardisation be used to improve bedside rounds?
- What concerns or potential barriers exist?

**Target commonly held beliefs with evidence**

- ‘Learners do not prefer bedside rounds’
  ○ Satisfaction often improves with increased exposure
  ○ Standardised practices may improve faculty member ratings by learners
  ○ Learners rate rotations implementing standardised rounds higher than controls
  ○ Learners recognise faculty members as being more invested in education
- ‘Patients do not like bedside rounds’
  ○ Patients perceive more time spent at bedside during standardised rounds
  ○ Patients overall satisfaction improves with standardised rounding practices
  ○ Standardisation increases the frequency of bedside rounds
- ‘I do not have time for bedside rounds, much less a standardised protocol’
  ○ Standardised rounds decrease the overall time spent on rounds
  ○ Improved timeliness of discharge orders with standardised rounds

**Assess response**

- Request feedback during session as to whether or not evidence presented is convincing
- What concerns still exist?
- Commit to collecting and distributing feedback from learners on educator practices
- Continue to engage with faculty members in order to address issues and promote change

We propose a session that begins by introducing faculty members to the data presented here, which supports the use of bedside rounds from the perspective of learners and patients. Faculty members might then benefit from a pragmatic discussion of the methods used in the papers identified here and, via small groups, attempt to distinguish potential components that might be incorporated into their own practices, as well as the potential limitations and difficulties that may come with such changes. Learning outcomes for these sessions include a recognition of some of the commonly cited barriers to bedside rounds and how standardised protocols might be used to mitigate or overcome perceived difficulties. For example, session leaders might elicit experiences or opinions regarding the timeliness of bedside rounds, and then provide the evidence included in this review detailing the improved timeliness of discharges, and the decreased time to complete bedside rounds overall, upon implementation of standardised protocols.

We have delineated what we believe to be the three most important aspects of a successful bedside rounds protocol above, but some faculty members may have more or less experience with rounds, and therefore more or less willingness to adopt certain practices, before any such session, and so we feel that these small group sessions may encourage near‐peer facilitation of uptake and further disrupt preconceived notions of bedside rounds.

A session such as this could easily be conducted within an hour and could take the form of a journal club style meeting, where the evidence for and against each of the studies, and their respective bedside rounds methods, could be argued. This might engender a sense of community practice among clinical educators and simultaneously present evidence and enable a more uniform style of rounds practices for the clinical environment.

Whichever strategies educators decide to implement for bedside rounds, it is important to note that flexibility will always be key in the fluid clinical care environment, where competing priorities are always in flux. Flexibility notwithstanding, we recommend that educators incorporate standardised strategies in their practice (pre‐round huddles, rounds at the bedside, inclusion of nursing staff) to attain the benefits of standardisation, with decreased time to complete rounds and with learners and patients being able to interact more efficiently and reliably if expectations are clearly established.

Flexibility notwithstanding, we recommend that educators incorporate standardised strategies in their practice …

### 4.2 Limitations

The limitations of this study are primarily related to the low number of studies included, leading to a low sample size, and the wide variation in methodology of each study. Our work here, however, constitutes a representation of the available literature on the standardisation of bedside rounds, and through it we have identified the major gaps and scarcity of data on this topic, reflecting the fragmented and hard‐to‐compare nature of the extant literature on bedside rounds.

## 5 CONCLUSION

In assessing the impact and practice of standardised bedside teaching rounds, this review highlights evidence that may dispel commonly held misconceptions regarding bedside rounds and provides educators tools to implement effective and practical teaching rounds. Through the implementation of standardised practices, clinical educators might improve patient satisfaction, learner satisfaction and the overall efficiency of clinical care.

Through the implementation of standardised practices, clinical educators might improve patient satisfaction …
